# An economical Nanopore sequencing assay for human papillomavirus (HPV) genotyping

**DOI:** 10.1186/s13000-020-00964-6

**Published:** 2020-05-06

**Authors:** Wai Sing Chan, Tsun Leung Chan, Chun Hang Au, Chin Pang Leung, Man Yan To, Man Kin Ng, Sau Man Leung, May Kwok Mei Chan, Edmond Shiu Kwan Ma, Bone Siu Fai Tang

**Affiliations:** Department of Pathology, Hong Kong Sanatorium & Hospital, Hong Kong, China

**Keywords:** Cervical cancer, HPV, Nanopore, NGS

## Abstract

**Background:**

Human papillomavirus (HPV) testing has been employed by several European countries to augment cytology-based cervical screening programs. A number of research groups have demonstrated potential utility of next-generation sequencing (NGS) for HPV genotyping, with comparable performance and broader detection spectrum than current gold standards. Nevertheless, most of these NGS platforms may not be the best choice for medium sample throughput and laboratories with less resources and space. In light of this, we developed a Nanopore sequencing assay for HPV genotyping and compared its performance with cobas HPV Test and Roche Linear Array HPV Genotyping Test (LA).

**Methods:**

Two hundred and one cervicovaginal swabs were routinely tested for Papanicolaou smear, cobas HPV Test and LA. Residual DNA was used for Nanopore protocol after routine testing. Briefly, HPV L1 region was amplified using PGMY and MGP primers, and PCR-positive specimens were sequenced on MinION flow cells (R9.4.1). Data generated in first 2 h were aligned with reference sequences from Papillomavirus Episteme database for genotyping.

**Results:**

Nanopore detected 96 HPV-positive (47.76%) and 95 HPV-negative (47.26%) specimens, with 10 lacking β-globin band and not further analyzed (4.98%). Substantial agreement was achieved with cobas HPV Test and LA (κ: 0.83–0.93). In particular, Nanopore appeared to be more sensitive than cobas HPV Test for HPV 52 (*n* = 7). For LA, Nanopore revealed higher concordance for high-risk (κ: 0.93) than non-high risk types (κ: 0.83), and with similar high-risk positivity in each cytology grading. Nanopore also provided better resolution for HPV 52 in 3 specimens co-infected with HPV 33 or 58, and for HPV 87 which was identified as HPV 84 by LA. Interestingly, Nanopore identified 5 additional HPV types, with an unexpected high incidence of HPV 90 (*n* = 12) which was reported in North America and Belgium but not in Hong Kong.

**Conclusions:**

We developed a Nanopore workflow for HPV genotyping which was economical (about USD 50.77 per patient specimen for 24-plex runs), and with comparable or better performance than 2 reference methods in the market. Future prospective study with larger sample size is warranted to further evaluate test performance and streamline the protocol.

## Introduction

Human papillomavirus (HPV) is generally accepted as the causative agent of cervical cancer (CC) [1], which was first unmasked by the landmark studies of Meisels and Fortin [2] and Purola and Savia [3]. Currently, there are 198 reference HPV types listed on Papillomavirus Episteme (PaVE) database, and at least 12 were classified as high-risk by World Health Organization (WHO) International Agency for Research on Cancer (IARC) Monographs Working Group [4–6]. HPV testing has been adopted by several European countries for primary CC screening, to augment cytology-based screening programs [7, 8]. A number of HPV assays are available commercially, which are mainly based on direct HPV genome detection, HPV DNA amplification and E6/ E7 mRNA detection [9]. Recent advent of next-generation sequencing (NGS) technologies has facilitated high throughput tools for infectious disease diagnostics and epidemiological research. Several research groups have explored utility of Illumina MiSeq and Ion Torrent platforms for HPV genotyping, with comparable sensitivity to well-established line blot assays and broader detection spectrum [10–12]. While the reagent cost is comparable to existing commercial assays for large sample batches, these NGS platforms may not be the best choice for medium sample throughput and laboratories with less resources and space. In this regard, portable Nanopore sequencers may allow more flexibility with shorter sequencing time and lower reagent cost. In light of this, we developed a Nanopore HPV genotyping protocol using 2 published primer sets, and compared its performance with 2 commercial HPV assays: cobas HPV Test and Roche Linear Array HPV Genotyping Test (LA).

## Methods

### Specimens

Two hundred and one cervicovaginal swabs were collected from March to July, 2019 in Hong Kong Sanatorium & Hospital. The swabs were preserved in SurePath preservative fluid (Becton, Dickson and Company, Sparks, MD, USA) and routinely tested for Papanicolaou smear (Pap smear, following The Bethesda System for reporting), cobas HPV Test and LA (Roche Diagnostics, Mannheim, Germany). Routine test results are shown in Table 1.

**Table 1:** Results of Pap smear, cobas HPV Test, Roche Linear Array HPV Genotyping Test, and Nanopore sequencing

Patient	Pap smear	Roche Linear Array		Cobas HPV	Nanopore (PGMY)		Nanopore (MGP)		Total HPV reads
		HR	Non-HR		HR	Non-HR	HR	Non-HR	
1	AGUS	Neg	Neg	Neg	Neg	Neg	Neg	Neg	ND
2	ASCH	52, 59	62	Other HR	59	Neg	59	90	4956
3	ASCUS	52	55	Neg	52	55	Neg	Neg	4262
4	ASCUS	Neg	Neg	Neg	Neg	Neg	Neg	Neg	ND
5	ASCUS	31, 33	54	Other HR	31, 33, 52	Neg	Neg	90	8973
6	ASCUS	Neg	Neg	Neg	Neg	Neg	Neg	Neg	ND
7	ASCUS	31	Neg	Other HR	Neg	Neg	31	Neg	1430
8	ASCUS	Neg	Neg	Neg	Neg	Neg	Neg	Neg	ND
9	ASCUS	Neg	81	Neg	Neg	81	Neg	81	48,477
10	ASCUS	18	Neg	18	18	Neg	18	Neg	16,206
11	ASCUS	Neg	Neg	Neg	Neg	Neg	Neg	Neg	ND
12	ASCUS	Neg	Neg	Neg	Neg	Neg	Neg	Neg	ND
13	ASCUS	52	53, 54	Other HR	52	44, 53, 74	52	74, 90	15,419
14	ASCUS	Neg	Neg	Neg	Neg	Neg	Neg	Neg	ND
15	ASCUS	Neg	Neg	Neg	Neg	Neg	Neg	Neg	ND
16	ASCUS	52	81	Neg	52	81	Neg	81	8873
17	ASCUS	52	54	Other HR	52	54	52	54	36,258
18	ASCUS	52, 59	11	Other HR	52, 59	11	52, 59	11	44,702
19	ASCUS	Neg	Neg	Neg	PCR inhibition				
20	ASCUS	Neg	Neg	Neg	Neg	Neg	Neg	Neg	7
21	ASCUS	Neg	Neg	Neg	Neg	Neg	Neg	Neg	ND
22	ASCUS	Neg	Neg	Neg	Neg	Neg	Neg	Neg	ND
23	ASCUS	39	61, 72	Other HR	39	61, 72	39	87	1624
24	ASCUS	66	Neg	Other HR	66	Neg	66	Neg	10,383
25	ASCUS	68	61	Other HR	Neg	61	Neg	61	10,644
26	ASCUS	Neg	Neg	Neg	Neg	Neg	Neg	90	541
27	ASCUS	52	Neg	Neg	52	Neg	Neg	87	3614
28	ASCUS	Neg	62	Neg	Neg	62	Neg	62	45
29	ASCUS	Neg	Neg	Neg	Neg	Neg	Neg	Neg	ND
30	ASCUS	35	Neg	Other HR	35	Neg	35	Neg	1641
31	ASCUS	Neg	Neg	Neg	Neg	Neg	Neg	Neg	ND
32	ASCUS	52	Neg	Other HR	52	Neg	52	Neg	399
33	ASCUS	Neg	Neg	Neg	Neg	Neg	Neg	Neg	ND
34	ASCUS	51	84	Other HR	51	Neg	Neg	Neg	1853
35	ASCUS	Neg	Neg	Neg	Neg	74	Neg	74	11,499
36	ASCUS	Neg	Neg	Neg	Neg	Neg	Neg	Neg	93
37	ASCUS	51	Neg	Other HR	51	Neg	51	Neg	2897
38	ASCUS	Neg	40, 55, 83	Neg	Neg	40, 55, 83	Neg	40, 55, 83	47,736
39	ASCUS	Neg	Neg	Neg	Neg	Neg	Neg	Neg	ND
40	ASCUS	58	53, 55, 62	Other HR	52, 58	53, 55, 62, 74	52	53, 62, 74	42,106
41	ASCUS	52	42, 73	Other HR	52	42, 73	52	42, 73	15,778
42	ASCUS	Neg	Neg	Neg	Neg	Neg	Neg	Neg	116
43	HSIL	16	Neg	16	16	Neg	16	Neg	15,918
44	HSIL	16	Neg	16	16	Neg	16	Neg	34,654
45	HSIL	59	Neg	Other HR	59	Neg	59	Neg	15,381
46	HSIL	31, 58	Neg	Other HR	31, 58	Neg	31, 58	Neg	3367
47	LSIL	52, 68	84	Other HR	52, 68	84	52, 68	84, 90	24,366
48	LSIL	66	84	Other HR	66	44, 84	66	44	57,206
49	LSIL	52	Neg	Neg	52	Neg	52	Neg	14,516
50	LSIL	Neg	40, 53	Neg	Neg	40, 53	Neg	40, 53	9265
51	LSIL	52	11, 81	Other HR	52	11, 81	52	11, 43, 81	29,748
52	LSIL	66	Neg	Other HR	66	Neg	66	Neg	40,328
53	LSIL	51	Neg	Other HR	51	Neg	51	43, 90	4454
54	LSIL	16, 51, 56	54, 62, 81	16, other HR	16, 51, 56	54, 62, 81	16, 51	40, 62, 81	20,455
55	LSIL	56	53	Other HR	56	53	56	53	28,377
56	LSIL	Neg	Neg	Neg	Neg	Neg	Neg	Neg	ND
57	LSIL	66	54, 55, 81	Other HR	66	54, 55, 81	66	55, 81, 90	25,606
58	LSIL	52	Neg	Neg	52	42	52	90	15,103
59	LSIL	59	Neg	Other HR	59	Neg	Neg	Neg	11,235
60	LSIL	59	89	Neg	59	89	Neg	89	67,220
61	LSIL	56	82	Other HR	56	82	56	43, 82	42,160
62	LSIL	52	Neg	Other HR	52	Neg	52	Neg	39,323
63	LSIL	33, 51	Neg	Other HR	33, 51	44	51	44	19,704
64	LSIL+ ASCH	51	Neg	Other HR	51	Neg	51	Neg	4621
65	NIL	16	Neg	16	16	Neg	16	Neg	1958
66	NIL	Neg	Neg	Neg	Neg	Neg	Neg	Neg	ND
67	NIL	Neg	Neg	Neg	Neg	Neg	Neg	Neg	ND
68	NIL	Neg	Neg	Neg	59	Neg	59	Neg	2455
69	NIL	Neg	Neg	Neg	Neg	87	Neg	87	8775
70	NIL	Neg	Neg	Neg	Neg	Neg	Neg	Neg	ND
71	NIL	Neg	Neg	Neg	Neg	Neg	Neg	Neg	ND
72	NIL	Neg	Neg	Neg	Neg	Neg	Neg	Neg	ND
73	NIL	Neg	Neg	Neg	Neg	Neg	Neg	Neg	ND
74	NIL	58	Neg	Other HR	58	Neg	52, 58	62	8619
75	NIL	58	Neg	Other HR	58	Neg	58	Neg	13,149
76	NIL	Neg	Neg	Neg	Neg	Neg	Neg	Neg	ND
77	NIL	Neg	Neg	Neg	Neg	Neg	Neg	Neg	ND
78	NIL	Neg	Neg	Neg	Neg	Neg	Neg	90	2289
79	NIL	56	70	Other HR	Neg	44, 70	56	44, 70	7855
80	NIL	Neg	Neg	Neg	PCR inhibition				
81	NIL	Neg	Neg	Neg	Neg	Neg	Neg	Neg	74
82	NIL	Neg	42	Neg	Neg	Neg	Neg	42	1406
83	NIL	Neg	Neg	Neg	Neg	74	Neg	74	7441
84	NIL	Neg	Neg	Neg	Neg	Neg	Neg	Neg	ND
85	NIL	Neg	82	Neg	Neg	82	Neg	82	1162
86	NIL	Neg	62	Neg	Neg	62	Neg	62	65,368
87	NIL	Neg	Neg	Neg	Neg	Neg	Neg	Neg	ND
88	NIL	Neg	Neg	Neg	Neg	Neg	Neg	Neg	ND
89	NIL	Neg	Neg	Neg	Neg	Neg	Neg	Neg	ND
90	NIL	Neg	Neg	Neg	Neg	Neg	Neg	Neg	142
91	NIL	39, 52	Neg	Other HR	52	Neg	52	90	15,703
92	NIL	68	Neg	Other HR	68	42	68	Neg	19,777
93	NIL	Neg	Neg	Neg	Neg	Neg	Neg	Neg	ND
94	NIL	Neg	Neg	Neg	Neg	Neg	Neg	Neg	ND
95	NIL	Neg	Neg	Neg	Neg	Neg	Neg	Neg	ND
96	NIL	52	Neg	Neg	52	Neg	52	Neg	5242
97	NIL	Neg	Neg	Neg	Neg	Neg	Neg	Neg	ND
98	NIL	Neg	Neg	Neg	Neg	Neg	Neg	Neg	ND
99	NIL	Neg	Neg	Neg	Neg	Neg	Neg	Neg	41
100	NIL	52	Neg	Other HR	52	Neg	52	Neg	24,478
101	NIL	Neg	61	Neg	PCR inhibition				
102	NIL	Neg	Neg	Neg	Neg	Neg	Neg	Neg	72
103	NIL	39	Neg	Neg	Neg	Neg	Neg	Neg	ND
104	NIL	Neg	62, 84	Neg	Neg	62	Neg	62	3589
105	NIL	Neg	71	Neg	Neg	Neg	Neg	Neg	ND
106	NIL	Neg	Neg	Neg	Neg	Neg	Neg	Neg	ND
107	NIL	52	62	Other HR	52	44, 53, 62	52	44	18,086
108	NIL	Neg	Neg	Neg	Neg	Neg	Neg	Neg	ND
109	NIL	Neg	Neg	Neg	Neg	Neg	Neg	Neg	ND
110	NIL	Neg	Neg	Neg	Neg	Neg	Neg	Neg	ND
111	NIL	Neg	84	Neg	Neg	Neg	Neg	Neg	ND
112	NIL	16, 52	Neg	16	16, 52	Neg	16	Neg	72,357
113	NIL	Neg	Neg	Neg	Neg	Neg	Neg	Neg	ND
114	NIL	Neg	55, 89	Neg	Neg	26, 55, 89	59	26, 55, 62, 89	8926
115	NIL	Neg	Neg	Neg	Neg	Neg	Neg	74	1586
116	NIL	Neg	81	Neg	Neg	Neg	Neg	Neg	ND
117	NIL	Neg	Neg	Neg	Neg	Neg	Neg	Neg	ND
118	NIL	Neg	6, 62	Neg	Neg	6, 62	Neg	6, 62	9414
119	NIL	Neg	Neg	Neg	Neg	Neg	Neg	Neg	ND
120	NIL	Neg	54	Neg	Neg	Neg	Neg	Neg	ND
121	NIL	Neg	Neg	Neg	PCR inhibition				
122	NIL	Neg	Neg	Neg	Neg	Neg	Neg	Neg	8
123	NIL	68	Neg	Other HR	Neg	Neg	Neg	Neg	ND
124	NIL	Neg	81	Neg	Neg	81	Neg	81	8735
125	NIL	Neg	84	Neg	Neg	Neg	Neg	87	1025
126	NIL	Neg	Neg	Neg	Neg	Neg	Neg	90	1719
127	NIL	Neg	Neg	Neg	Neg	Neg	Neg	Neg	ND
128	NIL	Neg	Neg	Neg	Neg	Neg	Neg	Neg	ND
129	NIL	Neg	Neg	Neg	Neg	Neg	Neg	Neg	10
130	NIL	Neg	Neg	Neg	Neg	Neg	Neg	Neg	ND
131	NIL	Neg	84	Neg	Neg	Neg	Neg	Neg	ND
132	NIL	Neg	Neg	Neg	Neg	Neg	Neg	Neg	ND
133	NIL	59	62, 71	Other HR	Neg	Neg	Neg	Neg	30
134	NIL	Neg	Neg	Neg	Neg	Neg	Neg	Neg	ND
135	NIL	Neg	Neg	Neg	Neg	Neg	Neg	Neg	522
136	NIL	Neg	Neg	Neg	Neg	Neg	Neg	Neg	ND
137	NIL	51	84	Other HR	PCR inhibition				
138	NIL	39	Neg	Other HR	39	Neg	39	Neg	19,305
139	NIL	Neg	Neg	Neg	Neg	Neg	Neg	Neg	195
140	NIL	Neg	Neg	Neg	Neg	Neg	Neg	Neg	ND
141	NIL	Neg	Neg	Neg	Neg	Neg	Neg	Neg	23
142	NIL	Neg	Neg	Neg	Neg	Neg	Neg	Neg	ND
143	NIL	Neg	42, 81	Neg	Neg	40, 74, 81	Neg	40, 74, 81, 87	19,118
144	NIL	Neg	Neg	Neg	Neg	Neg	Neg	Neg	ND
145	NIL	Neg	Neg	Neg	Neg	Neg	Neg	Neg	ND
146	NIL	Neg	Neg	Neg	Neg	Neg	Neg	Neg	ND
147	NIL	Neg	Neg	Neg	Neg	Neg	Neg	Neg	40
148	NIL	59	Neg	Neg	59	Neg	Neg	Neg	12,681
149	NIL	Neg	Neg	Neg	Neg	Neg	Neg	Neg	14
150	NIL	Neg	Neg	Neg	PCR inhibition				
151	NIL	Neg	Neg	Neg	Neg	Neg	Neg	Neg	79
152	NIL	Neg	62	Neg	Neg	62	Neg	62	14,353
153	NIL	Neg	Neg	Neg	Neg	Neg	Neg	Neg	ND
154	NIL	Neg	Neg	Neg	Neg	Neg	Neg	Neg	ND
155	NIL	Neg	Neg	Neg	Neg	Neg	Neg	Neg	ND
156	NIL	52	54	Neg	52	54	52	54	18,397
157	NIL	39, 52	53, 61	Other HR	39	53, 61	39	53, 61	20,332
158	NIL	Neg	Neg	Neg	Neg	Neg	Neg	Neg	ND
159	NIL	Neg	Neg	Neg	Neg	Neg	Neg	Neg	60
160	NIL	Neg	Neg	Neg	PCR inhibition				
161	NIL	Neg	62	Neg	Neg	62	Neg	62	13,545
162	NIL	Neg	Neg	Neg	Neg	74	Neg	74	4514
163	NIL	Neg	62	Neg	Neg	62	Neg	62	11,894
164	NIL	Neg	Neg	Neg	Neg	Neg	Neg	Neg	ND
165	NIL	59	Neg	Neg	PCR inhibition				
166	NIL	Neg	Neg	Neg	Neg	Neg	Neg	Neg	ND
167	NIL	39	Neg	Other HR	39	Neg	39	Neg	52,831
168	NIL	Neg	Neg	Neg	Neg	Neg	Neg	Neg	ND
169	NIL	Neg	Neg	Neg	Neg	Neg	Neg	Neg	ND
170	NIL	66	Neg	Other HR	66	Neg	66	Neg	54,943
171	NIL	Neg	Neg	Neg	Neg	Neg	Neg	Neg	ND
172	NIL	Neg	Neg	Neg	Neg	Neg	Neg	Neg	ND
173	NIL	Neg	Neg	Neg	Neg	Neg	Neg	Neg	ND
174	NIL	66	Neg	Other HR	66	Neg	66	Neg	57,791
175	NIL	Neg	54	Neg	Neg	54	Neg	54	23,583
176	NIL	Neg	Neg	Neg	PCR inhibition				
177	NIL	16	62	16	Neg	53, 62	16	62	28,181
178	NIL	Neg	Neg	Neg	Neg	Neg	Neg	Neg	206
179	NIL	Neg	Neg	Neg	Neg	Neg	Neg	Neg	ND
180	NIL	Neg	Neg	Neg	Neg	Neg	Neg	Neg	ND
181	NIL	51, 66	Neg	Other HR	51, 66, 68	Neg	51, 66, 68	Neg	6952
182	NIL	16, 51, 58	61	Other HR	58	61	Neg	61	5737
183	NIL	Neg	Neg	Neg	Neg	Neg	Neg	Neg	ND
184	NIL	58	Neg	Other HR	58	Neg	58	Neg	43,034
185	NIL	58	70, 89	Other HR	58	70, 89	58	89	33,842
186	ND	Neg	Neg	Neg	Neg	Neg	Neg	Neg	414
187	ND	Neg	Neg	Neg	Neg	Neg	Neg	Neg	ND
188	ND	16	Neg	16	16	Neg	16	Neg	96,549
189	ND	Neg	Neg	Neg	Neg	Neg	Neg	Neg	ND
190	ND	Neg	Neg	Neg	Neg	Neg	Neg	Neg	ND
191	ND	56	Neg	Other HR	56	Neg	56	Neg	18,782
192	ND	51	Neg	Other HR	51	Neg	51	Neg	6020
193	ND	Neg	62	Neg	Neg	62	Neg	62	20,373
194	ND	Neg	Neg	Neg	Neg	Neg	Neg	Neg	ND
195	ND	52, 59	Neg	Other HR	52, 59	Neg	59	Neg	11,926
196	ND	59	Neg	Other HR	59	Neg	59	Neg	24,045
197	ND	52, 59	54, 70	Other HR	52, 59	70	52, 59	70, 90	46,523
198	ND	56, 66	53, 61, 84	Other HR	66	32, 53, 61, 84	56	32, 53, 61, 84	62,600
199	ND	Neg	62	Neg	Neg	Neg	Neg	Neg	ND
200	ND	Neg	53, 54, 81, 83	Neg	Neg	53, 54, 83	Neg	53, 81, 83	32,868
201	ND	Neg	Neg	Neg	PCR inhibition				

*AGUS* Atypical glandular cells of undetermined significance, *ASCH* Atypical squamous cells – cannot exclude HSIL, *ASCUS* Atypical squamous cells of undetermined significance, *HR* High-risk, *HSIL* High-grade squamous intraepithelial lesion, *LSIL* Low-grade squamous intraepithelial lesion, *ND* Pap smear/ MinION sequencing not done, *Neg* Negative, *NIL* normal cytology

### DNA extraction

DNA extraction and cobas HPV Test were performed using cobas 4800 system (Roche Diagnostics, Rotkreuz, Switzerland). Briefly, 500 μL of cervicovaginal specimen was added to 500 μL of sample preparation buffer and heated at 120 °C for 20 min. The mixture was brought to ambient temperature for 10 min and processed on cobas × 480 using ‘high-risk HPV DNA PCR’ protocol. Real-time polymerase chain reaction (PCR) was performed on cobas z 480. Fifty microliter of DNA extract was used for LA according to manufacturer’s recommendations. Residual DNA was used for Nanopore protocol after routine testing.

### HPV PCR

For each specimen, L1 region of HPV genome was amplified in 2 separate PCRs using PGMY and MGP primer sets [13, 14]. Primer sequences and cycling conditions are shown in Tables 2 and 3. Human β-globin gene was used as inhibition control and contamination was monitored by negative extraction control. Five microliter of each PCR amplicon was electrophoresized in 2% agarose gel (Invitrogen, Carlsbad, CA, USA) and analyzed. PCR-positive specimens were sequenced using Nanopore MinION.

**Table 2:** Primer sequences

Primer	5′ to 3′ sequence	References
**PGMY PCR**		
PGMY11-A	GCA CAG GGA CAT AAC AAT GG	[13]
PGMY11-B	GCG CAG GGC CAC AAT AAT GG	
PGMY11-C	GCA CAG GGA CAT AAT AAT GG	
PGMY11-D	GCC CAG GGC CAC AAC AAT GG	
PGMY11-E	GCT CAG GGT TTA AAC AAT GG	
PGMY09-F	CGT CCC AAA GGA AAC TGA TC	
PGMY09-G	CGA CCT AAA GGA AAC TGA TC	
PGMY09-H	CGT CCA AAA GGA AAC TGA TC	
PGMY09-I	G CCA AGG GGA AAC TGA TC	
PGMY09-J	CGT CCC AAA GGA TAC TGA TC	
PGMY09-K	CGT CCA AGG GGA TAC TGA TC	
PGMY09-L	CGA CCT AAA GGG AAT TGA TC	
PGMY09-M	CGA CCT AGT GGA AAT TGA TC	
PGMY09-N	CGA CCA AGG GGA TAT TGA TC	
PGMY09-P	G CCC AAC GGA AAC TGA TC	
PGMY09-Q	CGA CCC AAG GGA AAC TGG TC	
PGMY09-R	CGT CCT AAA GGA AAC TGG TC	
HMB01	GCG ACC CAA TGC AAA TTG GT	
Human β-globin forward	GAAGAGCCAAGGACAGGTAC	[15]
Human β-globin reverse	GGAAAATAGACCAATAGGCAG	
**MGP PCR**		
MGPA	ACGTTGGATGTTTGTTACTGTGGTGGATACTAC	[16]
MGPB	ACGTTGGATGTTTGTTACCGTTGTTGATACTAC	
MGPC	ACGTTGGATGTTTGTTACTAAGGTAGATACCACTC	
MGPD	ACGTTGGATGTTTGTTACTGTTGTGGATACAAC	
MGP31	ACGTTGGATGTTTGTTACTATGGTAGATACCACAC	
MGPG	ACGTTGGATGGAAAAATAAACTGTAAATCATATTCCT	
MGPH	ACGTTGGATGGAAAAATAAATTGTAAATCATACTC	
MGPI	ACGTTGGATGGAAATATAAATTGTAAATCAAATTC	
MGPJ	ACGTTGGATGGAAAAATAAACTGTAAATCATATTC	
MGP18	ACGTTGGATGGAAAAATAAACTGCAAATCATATTC

**Table 3:** Master mix constituents and PCR conditions

PGMY PCR		
***Master mix constituents (for single reaction)***		
**Reagent**		**Volume/μL**
10X PCR buffer II (Applied Biosystems)		5
25 mM MgCl_2_ (Applied Biosystems)		3
PGMY primer mix (10 μM)		1
Human β-globin primer mix (5 μM)		1
10 mM dNTPs (Roche)		1
5 M betaine (Sigma)		10
AmpliTaq Gold DNA Polymerase (Applied Biosystems)		0.25
Molecular grade water (Sigma)		23.75
DNA		5
***PCR conditions***		
**Temperature/**^**o**^**C**	**Time**	**No. of cycles**
95	9 min	1
95	1 min	40 (50% ramp)
55	1 min	
72	1 min	
72	5 min	1
15	Hold	/
MGP PCR		
***Master mix constituents (for single reaction)***		
**Reagent**		**Volume/μL**
10X PCR buffer II (Applied Biosystems)		2.5
25 mM MgCl_2_ (Applied Biosystems)		1.5
MGP primer mix (10 μM)		0.5
10 mM dNTPs (Roche)		0.5
AmpliTaq Gold DNA Polymerase (Applied Biosystems)		0.1
Molecular grade water (Sigma)		14.9
DNA		5
***PCR conditions***		
**Temperature/**^**o**^**C**	**Time**	**No. of cycles**
95	10 min	1
95	30 s	5
42	30 s	
72	30 s	
95	30 s	45
64	30 s	
72	30 s	
72	5 min	1
15	Hold	/

### Nanopore sequencing library preparation

PGMY and MGP PCR amplicons of each positive specimen were pooled and purified using AMPure XP beads (Beckman-Coulter, Brea, CA, USA). Nanopore sequencing libraries were prepared from purified amplicons using Ligation Sequencing Kit 1D (SQK-LSK109) and PCR-free Native Barcoding Expansion Kit (EXP-NBD104/114) (Oxford Nanopore Technologies, Oxford, England). The barcoded libraries were loaded and sequenced on MinION flow cells (FLO-MIN106D R9.4.1, Oxford Nanopore Technologies, Oxford, England) after quality control runs.

### Data analysis

Data from first 2 h of sequencing runs was analyzed. FASTQ files generated by live basecalling (MinKNOW version 2.0) were demultiplexed using ‘FASTQ Barcoding’ workflow on EPI2ME (Oxford Nanopore Technologies, Oxford, England) with default minimum qscore of 7, ‘auto’ and ‘split by barcode’ options. FASTQ files of each specimen were concatenated into a single file and analyzed using a 2-step custom workflow on Galaxy bioinformatics platform. Briefly, FASTQ files were converted into FASTA format, followed by aligning sequences against HPV reference genomes from PaVE database using NCBI BLAST+ blastn (Galaxy version 1.1.1). PGMY and MGP reads were sorted based on sequence length and analyzed individually. Threshold of each run was derived from average number of background reads plus 10 standard deviations, which were calculated using interquartile rule, excluding first and last quartiles. A positive HPV call was based on either (1) the number of reads for a particular HPV type was above threshold, or (2) the specimen had the highest number of reads for a particular HPV type. All positive calls were further assessed by aligning FASTQ sequences against HPV reference genomes using minimap2 (Galaxy version 2.17 + galaxy0), and consensus sequences were built from BAM files using Unipro UGENE (version 1.29.0) for determining their percentage of identity to reference genomes.

## Results

As HPV 66 is categorized as ‘other high-risk’ by cobas HPV Test, all calculations were based on this grouping, albeit HPV 66 was found as a single infection in cancers with extreme rarity and re-classified as possible carcinogen (Group 2B) by IARC Monographs Working Group [6].

The results are summarized in Table 1. PCR was successful for 191 specimens (191/201, 95.02%), with 10 specimens (10/201, 4.98%) lacking β-globin band and therefore regarded as inappropriate for further analysis. Seventy-six specimens (76/201, 37.81%) were negative for both PGMY and MGP PCRs, and 115 (115/201, 57.21%) were positive for either of the two. PCR-positive specimens were sequenced on 10 MinION flow cells with 145–890 active pores, generating 31,748–525,880 HPV reads in first 2 h (Table 4). For the 115 specimens sequenced, 19 were negative (7–522 reads, 113 in average) and 96 were positive (45–96,549 reads, 20,158 in average) for HPV. Taken together, there were 95 HPV-negative (95/201, 47.26%) and 96 HPV-positive (96/201, 47.76%) specimens by Nanopore workflow.

**Table 4:** Details of Nanopore sequencing runs

Run	No. of active pores	Elapsed sequencing time	No. of HPV reads
1	611	2 h 11 min	60,976
2	458	1 h 59 min	246,521
3	690	2 h 1 min	279,520
4	467	2 h 5 min	111,885
5	462	2 h 5 min	31,748
6	247	2 h 3 min	113,521
7	330	2 h 5 min	111,702
8	753	2 h 1 min	478,711
9	145	1 h 59 min	207,094
10	890	1 h 59 min	525,880

Table 5 shows concordance of Nanopore workflow with cobas HPV Test and LA, which was based on the 37 HPV types detectable by LA. For cobas HPV Test, our workflow achieved 93.19, 93.19 and 81.94% for perfect, total and positive agreement, respectively, with Cohen’s kappa of 0.85. For LA, Nanopore achieved a perfect agreement of 83.77% for both high-risk and non-high risk HPVs. For high-risk types, total and positive agreement were 96.86 and 91.78%, respectively, with Cohen’s kappa of 0.93. For non-high risk types, total and positive agreement were 93.19 and 77.59%, respectively, with Cohen’s kappa of 0.83.

**Table 5:** Agreement between cobas HPV Test, Roche Linear Array HPV Genotyping Test (LA) and Nanopore

			Nanopore		Perfect agreement	Total agreement	Positive agreement	Cohen’s κ
			+	–				
**cobas HPV Test**	**+**		59	2	93.19%	93.19%	81.94%	0.85
	**–**		11	119				
**LA**	**HR**	**+**	67	4	83.77%	96.86%	91.78%	0.93
		**–**	2	118				
	**Non-HR**	**+**	45	10		93.19%	77.59%	0.83
		**–**	3	133				

Table 6 shows per-type concordance of Nanopore and LA. A total of 13 high-risk and 19 non-high risk HPV types were evaluated. Positive agreement for HPV 16 (*n* = 8) and 18 (*n* = 1) were 87.5 and 100%, respectively. Positive agreement was 75–100% for high-risk HPV 31, 33, 35, 39, 51, 52, 56, 58, 59 and 66, and 20% for HPV 68 (*n* = 5). For non-high risk HPVs, positive agreement was 37.5–100% for HPV 6, 11, 40, 42, 53, 54, 55, 61, 62, 70, 72, 73, 81, 82, 83, 84 and 89. There were 2 non-high risk types with 0% positive agreement (HPV 26 and 71). HPV 26 (*n* = 1) was only detected by Nanopore workflow, whereas HPV 71 (*n* = 2) was only detected by LA.

**Table 6:** Per HPV type positive agreement between Roche Linear Array Genotyping Test (LA) and Nanopore

HPV Genotypes		Number of specimens					Positive agreement
		Nanopore−/LA−/LA-	Nanopore +/LA-	Nanopore−/LA+	Nanopore+/LA+	Total	
**High-risk**	**16**	183	0	1	7	191	87.5%
	**18**	190	0	0	1	191	100%
	**31**	188	0	0	3	191	100%
	**33**	189	0	0	2	191	100%
	**35**	190	0	0	1	191	100%
	**39**	185	0	1	5	191	83.33%
	**51**	182	0	1	8	191	88.89%
	**52**	165	3	2	21	191	80.77%
	**56**	185	0	0	6	191	100%
	**58**	184	0	0	7	191	100%
	**59**	179	2	1	9	191	75%
	**66**	182	1	0	8	191	88.89%
	**68**	186	2	2	1	191	20%
**Non-high risk**	**6**	190	0	0	1	191	100%
	**11**	189	0	0	2	191	100%
	**26**	190	1	0	0	191	0%
	**40**	187	2	0	2	191	50%
	**42**	186	2	1	2	191	40%
	**53**	181	3	0	7	191	70%
	**54**	181	0	4	6	191	60%
	**55**	186	0	0	5	191	100%
	**61**	186	0	0	5	191	100%
	**62**	174	2	2	13	191	76.47%
	**70**	188	0	0	3	191	100%
	**71**	189	0	2	0	191	0%
	**72**	190	0	0	1	191	100%
	**73**	190	0	0	1	191	100%
	**81**	182	0	1	8	191	88.89%
	**82**	189	0	0	2	191	100%
	**83**	189	0	0	2	191	100%
	**84**	183	0	5	3	191	37.5%
	**89**	188	0	0	3	191	100%

Table 7 reveals the percentage of identity of Nanopore consensus sequences to HPV reference genomes. In general, Nanopore consensus sequences showed an average identity of 98% to the best matches, with an average difference of 15% from second BLAST hits.

**Table 7:** Percentage of identity of Nanopore consensus sequences to HPV reference genomes

Patient	Nanopore results	Best BLAST hit		Second BLAST hit		Difference
		HPV type	% identity	HPV type	% identity	
**2**	**59**	59	99%	18	77%	22%
	^**a**^**90**	90	97%	106	84%	15%
**3**	**52**	52	99%	58	80%	19%
	**55**	55	100%	44	93%	7%
**5**	**31**	31	98%	35	80%	18%
	**33**	33	99%	58	86%	13%
	^**a**^**52**	52	99%	58	80%	19%
	^**a**^**90**	90	97%	106	85%	12%
**7**	**31**	31	95%	35	79%	16%
**9**	**81**	81	99%	62	85%	14%
**10**	**18**	18	99%	45	85%	14%
**13**	^**a**^**44**	44	99%	55	92%	7%
	**52**	52	99%	58	80%	19%
	**53**	53	99%	30	85%	14%
	^**a**^**74**	74	99%	55	83%	16%
	^**a**^**90**	90	97%	106	85%	12%
**16**	**52**	52	99%	58	81%	18%
	**81**	81	99%	62	85%	14%
**17**	**52**	52	99%	58	80%	19%
	**54**	54	99%	45	74%	25%
**18**	**11**	11	99%	6	87%	12%
	**52**	52	99%	58	80%	19%
	**59**	59	99%	18	77%	22%
**23**	**39**	39	99%	70	81%	18%
	**61**	61	99%	mEV06c12b	83%	16%
	**72**	72	92%	mEV06c12b	89%	3%
	^**a**^**87**	87	98%	86	85%	13%
**24**	**66**	66	98%	56	84%	14%
**25**	**61**	61	99%	mEV06c12b	83%	16%
**26**	^**a**^**90**	90	97%	106	85%	12%
**27**	**52**	52	99%	58	80%	19%
	^**a**^**87**	87	98%	86	84%	14%
**28**	**62**	62	99%	81	84%	15%
**30**	**35**	35	98%	31	80%	18%
**32**	**52**	52	99%	58	81%	18%
**34**	**51**	51	99%	82	85%	14%
**35**	^**a**^**74**	74	99%	55	84%	15%
**37**	**51**	51	99%	82	85%	14%
**38**	**40**	40	99%	7	88%	11%
	**55**	55	99%	44	93%	6%
	**83**	83	99%	102	84%	15%
**40**	^**a**^**52**	52	99%	58	80%	19%
	**53**	53	98%	30	85%	13%
	**55**	55	100%	44	93%	7%
	**58**	58	99%	33	86%	13%
	**62**	62	99%	81	85%	14%
	^**a**^**74**	74	98%	55	84%	14%
**41**	**42**	42	98%	32	83%	15%
	**52**	52	100%	58	81%	19%
	**73**	73	99%	34	85%	14%
**43**	**16**	16	100%	35	78%	22%
**44**	**16**	16	99%	35	78%	21%
**45**	**59**	59	99%	18	76%	23%
**46**	**31**	31	98%	35	80%	18%
	**58**	58	99%	33	86%	13%
**47**	**52**	52	98%	58	80%	18%
	**68**	68	93%	39	81%	12%
	**84**	84	98%	87	84%	14%
	^**a**^**90**	90	97%	106	85%	12%
**48**	^**a**^**44**	44	99%	55	93%	6%
	**66**	66	98%	56	84%	14%
	**84**	84	99%	87	84%	15%
**49**	**52**	52	99%	58	80%	19%
**50**	**40**	40	98%	7	87%	11%
	**53**	53	98%	30	85%	13%
**51**	**11**	11	100%	6	87%	13%
	^**a**^**43**	43	95%	45	77%	18%
	**52**	52	99%	58	80%	19%
	**81**	81	99%	62	84%	15%
**52**	**66**	66	98%	56	83%	15%
**53**	^**a**^**43**	43	95%	45	78%	17%
	**51**	51	99%	82	84%	15%
	^**a**^**90**	90	97%	106	85%	12%
**54**	**16**	16	100%	35	78%	22%
	^**a**^**40**	40	93%	7	85%	8%
	**51**	51	99%	82	84%	15%
	**54**	54	99%	45	73%	26%
	**56**	56	90%	66	76%	14%
	**62**	62	99%	81	84%	15%
	**81**	81	99%	62	85%	14%
**55**	**53**	53	99%	56	79%	20%
	**56**	56	99%	66	84%	15%
**57**	**54**	54	87%	31	74%	13%
	**55**	55	100%	44	93%	7%
	**66**	66	98%	56	84%	14%
	**81**	81	99%	62	84%	15%
	^**a**^**90**	90	97%	106	85%	12%
**58**	^**a**^**42**	42	99%	32	84%	15%
	**52**	52	98%	58	80%	18%
	^**a**^**90**	90	97%	106	85%	12%
**59**	**59**	59	99%	18	77%	22%
**60**	**59**	59	99%	18	76%	23%
	**89**	89	99%	81	78%	21%
**61**	^**a**^**43**	43	96%	45	79%	17%
	**56**	56	97%	66	83%	14%
	**82**	82	99%	51	84%	15%
**62**	**52**	52	99%	58	80%	19%
**63**	**33**	33	99%	58	86%	13%
	^**a**^**44**	44	99%	55	93%	6%
	**51**	51	99%	82	83%	16%
**64**	**51**	51	99%	82	84%	15%
**65**	**16**	16	100%	35	78%	22%
**68**	^**a**^**59**	59	99%	18	77%	22%
**69**	^**a**^**87**	87	99%	86	86%	13%
**74**	^**a**^**52**	52	99%	58	81%	18%
	**58**	58	99%	33	86%	13%
	^**a**^**62**	62	99%	81	85%	14%
**75**	**58**	58	99%	33	85%	14%
**78**	^**a**^**90**	90	97%	106	85%	12%
**79**	^**a**^**44**	44	99%	55	92%	7%
	**56**	56	96%	66	84%	12%
	**70**	70	99%	39	81%	18%
**81**	^**a**^**74**	74	93%	55	81%	12%
**82**	**42**	42	95%	32	83%	12%
**83**	^**a**^**74**	74	97%	55	83%	14%
**85**	**82**	82	99%	51	84%	15%
**86**	**62**	62	99%	81	85%	14%
**91**	**52**	52	99%	58	80%	19%
	^**a**^**90**	90	97%	106	84%	13%
**92**	^**a**^**42**	42	93%	32	78%	15%
	**68**	68	92%	39	80%	12%
**96**	**52**	52	99%	58	80%	19%
**100**	**52**	52	99%	58	80%	19%
**104**	**62**	62	98%	81	85%	13%
**107**	^**a**^**44**	44	99%	55	93%	6%
	**52**	52	99%	58	81%	18%
	^**a**^**53**	53	100%	30	86%	14%
	**62**	62	99%	81	85%	14%
**112**	**16**	16	98%	58	78%	20%
	**52**	52	99%	58	81%	18%
**114**	^**a**^**26**	26	100%	69	83%	17%
	**55**	55	100%	44	93%	7%
	^**a**^**59**	59	99%	18	77%	22%
	^**a**^**62**	62	99%	81	85%	14%
	**89**	89	99%	81	77%	22%
**115**	^**a**^**74**	74	95%	55	83%	12%
**118**	**6**	6	99%	11	87%	12%
	**62**	62	99%	81	84%	15%
**124**	**81**	81	99%	62	85%	14%
**125**	^**a**^**87**	87	98%	86	85%	13%
**126**	^**a**^**90**	90	97%	106	85%	12%
**138**	**39**	39	99%	68	81%	18%
**143**	^**a**^**40**	40	99%	7	88%	11%
	^**a**^**74**	74	98%	55	84%	14%
	**81**	81	99%	62	84%	15%
	^**a**^**87**	87	97%	86	84%	13%
**148**	**59**	59	99%	18	77%	22%
**152**	**62**	62	98%	81	85%	13%
**156**	**52**	52	99%	58	81%	18%
	**54**	54	95%	6	74%	21%
**157**	**39**	39	94%	70	81%	13%
	**53**	53	96%	30	84%	12%
	**61**	61	99%	mEV06c12b	83%	16%
**161**	**62**	62	98%	81	83%	15%
**162**	^**a**^**74**	74	94%	55	85%	9%
**163**	**62**	62	99%	81	85%	14%
**167**	**39**	39	99%	70	81%	18%
**170**	**66**	66	98%	56	83%	15%
**174**	**66**	66	98%	56	83%	15%
**175**	**54**	54	99%	45	73%	26%
**177**	**16**	16	99%	35	80%	19%
	^**a**^**53**	53	99%	30	84%	15%
	**62**	62	99%	81	85%	14%
**181**	**51**	51	99%	82	85%	14%
	**66**	66	98%	56	83%	15%
	^**a**^**68**	68	98%	39	81%	17%
**182**	**58**	58	98%	33	87%	11%
	**61**	61	100%	mEV06c12b	83%	17%
**184**	**58**	58	99%	33	85%	14%
**185**	**58**	58	99%	33	85%	14%
	**70**	70	99%	39	81%	18%
	**89**	89	99%	81	78%	21%
**188**	**16**	16	100%	35	78%	22%
**191**	**56**	56	99%	66	83%	16%
**192**	**51**	51	98%	82	84%	14%
**193**	**62**	62	99%	81	85%	14%
**195**	**52**	52	99%	58	81%	18%
	**59**	59	99%	18	76%	23%
**196**	**59**	59	99%	18	77%	22%
**197**	**52**	52	100%	58	81%	19%
	**59**	59	99%	18	76%	23%
	**70**	70	99%	39	81%	18%
	^**a**^**90**	90	97%	106	85%	12%
**198**	^**a**^**32**	32	99%	42	84%	15%
	**53**	53	99%	30	86%	13%
	**56**	56	99%	66	84%	15%
	**61**	61	100%	mEV06c12b	83%	17%
	**66**	66	98%	56	83%	15%
	**84**	84	99%	87	84%	15%
**200**	**53**	53	98%	30	85%	13%
	**54**	54	99%	45	74%	25%
	**81**	81	99%	62	84%	15%
	**83**	83	95%	102	82%	13%
**Average % identity of the best hit**			**98%**	**Average difference**		**15%**

^a^ HPV types not detected by LA

Table 8 summarizes HPV status of each cytology grading. For high-grade and low-grade squamous intraepithelial lesion (HSIL and LSIL), nearly all specimens were positive for high-risk HPV (HSIL: 4/4, 100%; LSIL: 16/18, 88.89%). For atypical squamous/ glandular cells, about half of the specimens were positive for high-risk HPV (by LA: 19/41, 46.34%; by Nanopore: 18/41, 43.90%). For cases without observable abnormalities, 22.12% (25/113) and 21.24% (24/113) were positive for high-risk HPV by LA and Nanopore, respectively.

**Table 8:** Results of Pap smear, LA and Nanopore workflow. The calculations were based 176 quality control-valid specimens with Pap smear results available

Pap smear interpretation	HPV status	No. of specimens	
		LA	Nanopore
HSIL (*n* = 4)	HR/ HR + non-HR	4	4
	Non-HR only	0	0
	Negative	0	0
LSIL/ LSIL + ASCH (*n* = 18)	HR/ HR + non-HR	16	16
	Non-HR only	1	1
	Negative	1	1
AGUS/ ASCH/ ASCUS (*n* = 41)	HR/ HR + non-HR	19	18
	Non-HR only	3	6
	Negative	19	17
NIL (*n* = 113)	HR/ HR + non-HR	25	24
	Non-HR only	18	18
	Negative	70	71

## Discussion

Hong Kong has been one of the Asian regions with the lowest incidence and mortality rate of CC [16]. This might be attributable to the territory-wide cervical screening program implemented by Department of Health since 2004. The program is well-organized, which involves public education, regular cervical smear and follow-up service for eligible women, and a quality assurance mechanism on key components of the program [17]. Cytology is the mainstay of primary screening, and high-risk HPV testing may be performed for triage to colposcopy.

Cytology and HPV testing have their own value for CC screening. High quality cytology has high specificity for CC, but with lower sensitivity ranging from 50% suggested by cross-sectional studies to 75% estimated longitudinally [18]. For HPV testing, the sensitivity was reported to be about 10% higher than cytology, yet with lower specificity [18]. Complementary use of both tests could enhance the sensitivity approaching 100% with high specificity (92.5%) [19]. In fact, this combined approach has been adopted by several European countries and may become the future trend of primary CC screening in developed countries.

**Fig. 1:**
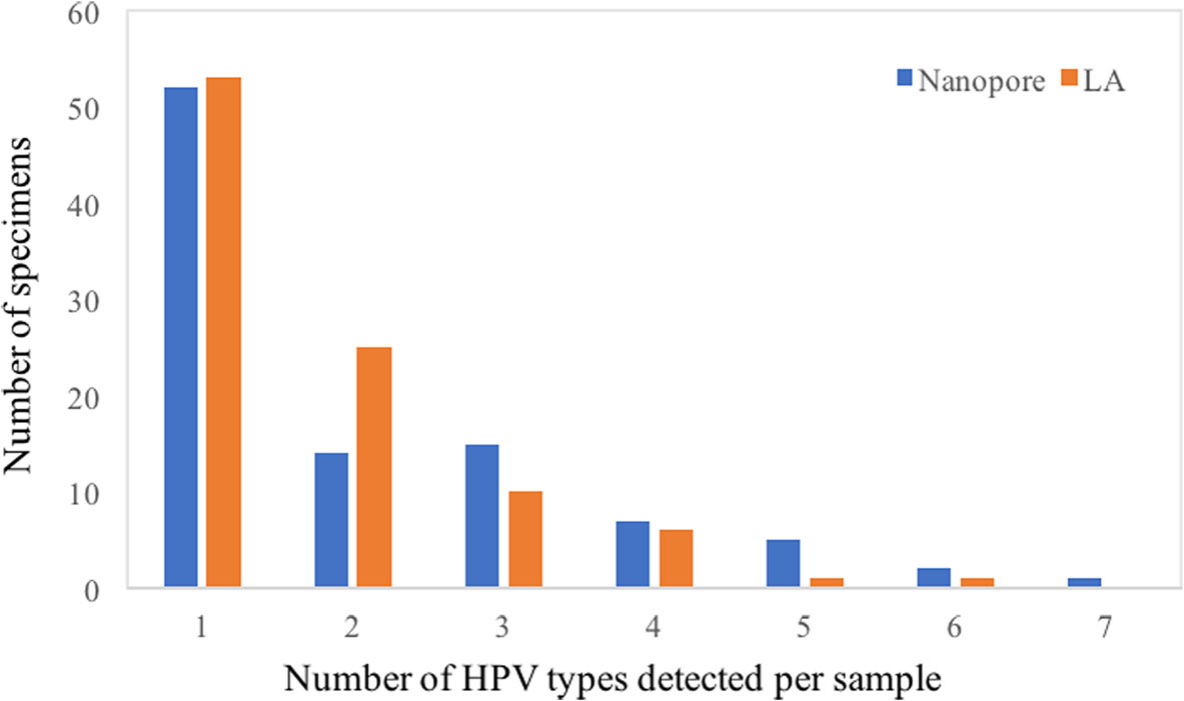
Number of HPV types detected per sample by Nanopore workflow and LA

**Fig. 2:**
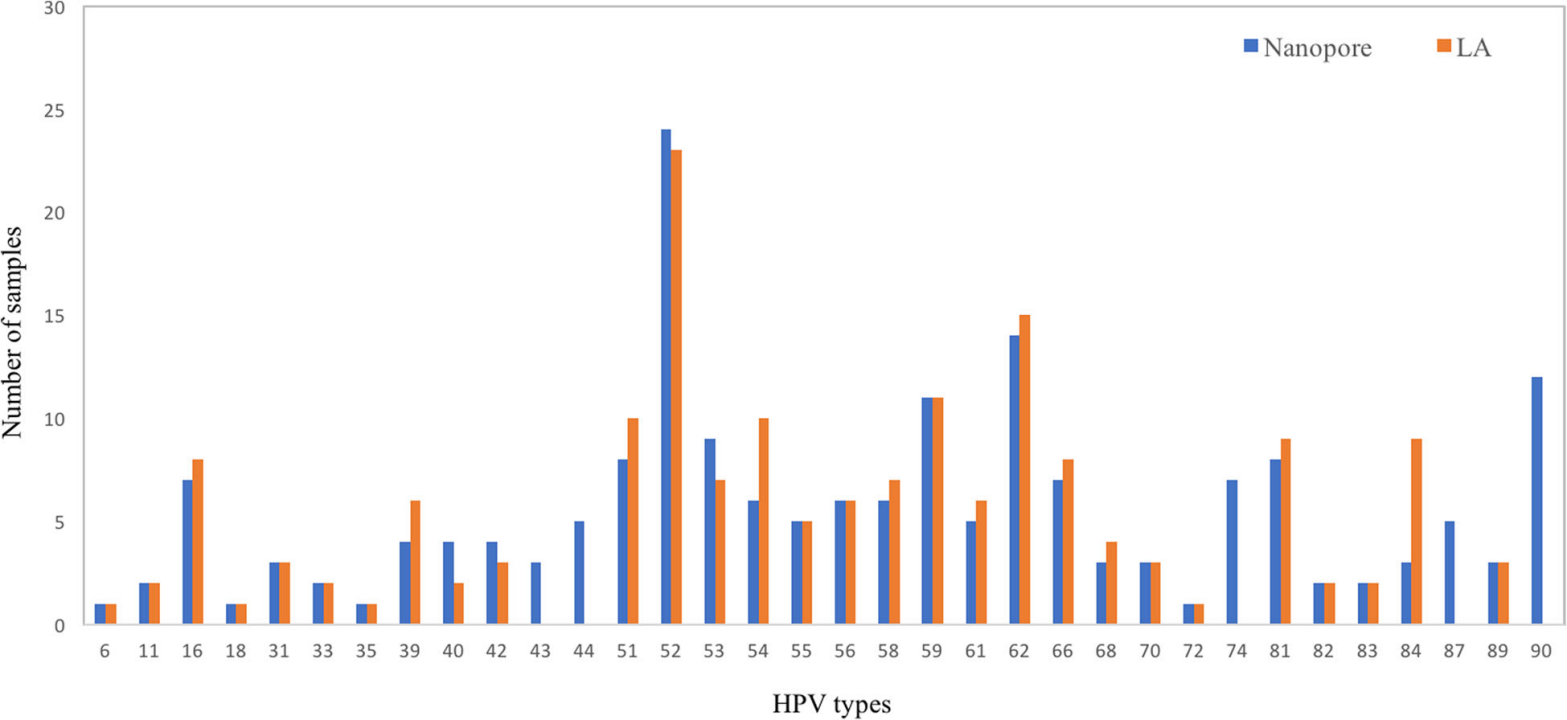
Diversity of HPV types detected by Nanopore workflow and LA

Compared with HPV assays in the market, HPV genotyping by NGS offers a broader detection spectrum which, despite minimal benefit of non-high risk HPV information for CC screening, may provide important etiologic clues for other HPV-associated infections and a more complete picture of HPV epidemiology. For the latter, Nanopore identified more HPV types per sample (Fig. 1) and 5 extra HPV types (HPV 43, 44, 74, 87 and 90, *n* = 34) not detectable by LA (Fig. 2), with an unexpected high incidence of HPV 90 (*n* = 12) which was reported in North America and Belgium but not in Hong Kong [20, 21]. Another advantage offered by NGS is its potential utility for simultaneous characterization of cervicovaginal microbiome, with its possible role in dysplasia and carcinogenesis revealed by accumulating research evidence [22–25]. These merits may facilitate a multifaceted approach for evaluation of woman health in near feature.

In general, Nanopore had substantial agreement with cobas HPV Test and LA. Compared with cobas HPV Test, Nanopore appeared to be more sensitive for HPV 52 (*n* = 7) and 59 (*n* = 4), with 81.82% (9/11) of these discrepant results matched with LA. Compared with LA, concordance for high-risk HPV was higher than non-high risk types. Among the 37 discrepant results, 22 were false negatives by Nanopore and 15 were not detected by LA.

For the false negatives by Nanopore, more than half (12/22, 54.55%) were mixed infections, and similar finding was reported by other research groups using HPV consensus primers for NGS-based genotyping [10, 11]. Other possible causes of false negatives included (1) low viral load, as evident by Specimen 182, from which HPV 16 was missed by both Nanopore and cobas HPV Test; (2) substantial difference in DNA input (50 μL for LA versus 5 μL for PGMY/ MGP PCR), as well as (3) lower sensitivity due to reduced magnesium chloride concentration of PGMY PCR (from 4 mM to 1.5 mM), which was fine-tuned for minimal non-specific amplification.

For the 15 HPV types missed by LA, the average identity of Nanopore consensus sequences was 98.27% with an average difference of 16% from second BLAST hits (Table 7). As distinct HPV types generally have more than 10% difference in L1 sequence [26, 27], it appeared that the discrepant positive calls were less likely caused by high sequencing error rate of Nanopore. More specifically, 5 of these positive calls were identified solely by MGP PCR (5/15, 33.33%), 5 detected by PGMY PCR only (5/15, 33.33%), and 5 by both PCRs (5/15, 33.33%). These revealed differential sensitivities of PGMY and MGP PCR primers, which might complement with each other and enhance overall performance of the Nanopore assay. On the other hand, Nanopore sequencing might improve the resolution of genotyping, which might not be attained by line blot method due to cross-hybridization of certain probes. For instance, Nanopore identified HPV 52 in Specimen 5, 40 and 74, which could not be confirmed by LA due to cross-hybridization with HPV 33 and 58, respectively. Another example was Specimen 125, which was HPV 84-positive by LA and HPV 87-positive by Nanopore. From literature, Artaza-Irigaray and colleagues reported cross-hybridization between these 2 HPV types by LA, with 11.5% of HPV 84-positive cervical specimens by LA were actually HPV 87-positive by NGS [28].

The Nanopore method and LA revealed very similar high-risk HPV positivity in each cytology grading. The goal of combined cytology-HPV testing approach is to enhance cost effectiveness of CC screening. While minimizing unnecessary referral for colposcopy, HPV genotyping may identify high-risk individuals before observable cytological abnormalities, for instance, the 4 HPV 16-positive patients without abnormal cytology findings in this study. This may facilitate an early detection approach for cancer prevention.

Our study had several limitations. First, the sample size of certain HPV types, for example, HPV 18 (*n* = 1), was less satisfactory for evaluating type-specific performance. Second, as residual DNA was used after routine testing, DNA input for PGMY and MGP PCRs was constrained which might lower the sensitivity. In addition, as flow cells with suboptimal number of active pores were used, sequencing time and depth might be further improved if new flow cells were used.

## Conclusions

We developed a Nanopore workflow for HPV genotyping, with performance comparable to or better than 2 reference methods in the market. Our method was economical, with a reagent cost of about USD 50.77 per patient specimen for 24-plex runs, which was competitive when compared to an average price of USD 106.14 (from 4 randomly-selected laboratories) for HPV genotyping referral service in our region (Table 9). The protocol was also straightforward with reasonable turnaround time of about 12 h from samples to answers. The small size and portability of MinION sequencers may well suit remote or resource-limited laboratories with constraints in space. Future prospective study with larger sample size is warranted to further evaluate test performance and streamline the protocol. As LA was discontinued in Hong Kong, the Nanopore workflow described here may provide an economical option for broad-range HPV genotyping.

**Table 9:** Comparison of estimated reagent cost of Nanopore workflow (24-plex) and randomly-selected prices of HPV genotyping referral service in Hong Kong

***This study***		
Procedure	Number of specimens	Cost
DNA extraction and PCRs	201 patients +20 controls = 221	USD 20.02 × 221 reactions = USD 4424.42
Nanopore sequencing	115 patients / 24 = at least 5 runs N = 120 for 1 positive control per run	USD 1155.94 × 5 runs = USD 5779.70
**Cost per patient specimen**		(4424.42 + 5779.70) / 201 = **USD 50.77**
***Referral service (transportation cost not included)***		
Lab A		USD 77.19
Lab B		USD 124.79
Lab C		USD 101.63
Lab D		USD 120.93
**Average**		**USD 106.14**

## Data Availability

The datasets used and/or analyzed during the current study are available from the corresponding author on reasonable request.

## Abbreviations

CC: Cervical cancer
HPV: Human papillomavirus
HSIL: High-grade squamous intraepithelial lesion
IARC: International Agency for Research on Cancer (IARC)
LA: Roche Linear Array HPV Genotyping Test
LSIL: Low-grade squamous intraepithelial lesion
NGS: Next-generation sequencing
Pap smear: Papanicolaou smear
PCR: Polymerase chain reaction
WHO: World Health Organization
